# Bayesian Machine Learning for Efficient Minimization of Defects in ALD Passivation Layers

**DOI:** 10.1021/acsami.1c14586

**Published:** 2021-11-04

**Authors:** Gül Dogan, Sinan O. Demir, Rico Gutzler, Herbert Gruhn, Cem B. Dayan, Umut T. Sanli, Christian Silber, Utku Culha, Metin Sitti, Gisela Schütz, Corinne Grévent, Kahraman Keskinbora

**Affiliations:** † Robert Bosch GmbH, Automotive Electronics, Postfach 13 42, 72703 Reutlingen, Germany; ‡ Max Planck Institute for Intelligent Systems, Heisenbergstr 3, 70569 Stuttgart, Germany; § Max Planck Institute for Solid State Research, Heisenbergstr 1, 70569 Stuttgart, Germany; ∥ Robert Bosch GmbH, Corporate Sector Research and Advance Engineering , Robert-Bosch-Campus1, 71272 Stuttgart, Germany

**Keywords:** atomic layer deposition, copper, defect density, wet etching, Bayesian optimization

## Abstract

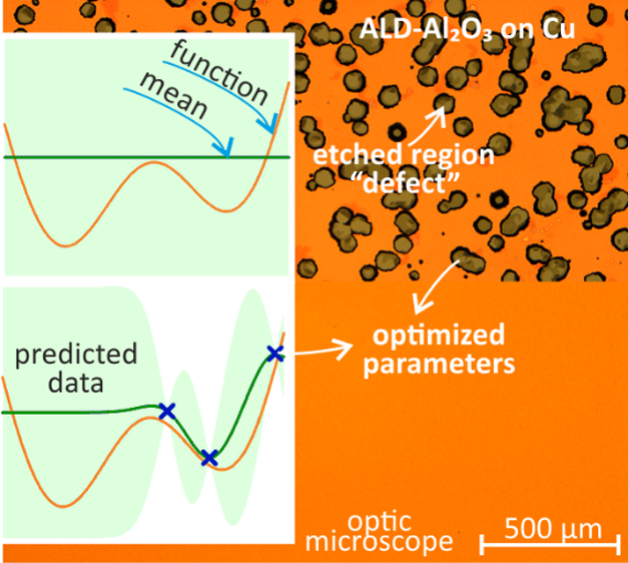

Atomic layer deposition (ALD) is an enabling technology for encapsulating sensitive materials owing to its high-quality, conformal coating capability. Finding the optimum deposition parameters is vital to achieving defect-free layers; however, the high dimensionality of the parameter space makes a systematic study on the improvement of the protective properties of ALD films challenging. Machine-learning (ML) methods are gaining credibility in materials science applications by efficiently addressing these challenges and outperforming conventional techniques. Accordingly, this study reports the ML-based minimization of defects in an ALD-Al_2_O_3_ passivation layer for the corrosion protection of metallic copper using Bayesian optimization (BO). In all experiments, BO consistently minimizes the layer defect density by finding the optimum deposition parameters in less than three trials. Electrochemical tests show that the optimized layers have virtually zero film porosity and achieve five orders of magnitude reduction in corrosion current as compared to control samples. Optimized parameters of surface pretreatment using Ar/H_2_ plasma, the deposition temperature above 200 °C, and 60 ms pulse time quadruple the corrosion resistance. The significant optimization of ALD layers presented in this study demonstrates the effectiveness of BO and its potential outreach to a broader audience, focusing on different materials and processes in materials science applications.

## Introduction

The utilization of machine-learning (ML)-based approaches has recently gained significant momentum in addressing the challenging problems in materials science and engineering.^1^ Their computational power is now outperforming the conventional optimization and simulation tools and allowing the improvement of complex processes in materials science.^2^ From these processes, the discovery of novel materials and the prediction of material properties have been the most active fields of research.^3,4^ In these application areas, ML methods efficiently employ the existing data to test new parameters that may yield novel material designs and properties. However, materials science applications can further take advantage of ML methods. In addition to the exploration of new materials, complex material fabrication processes (e.g., thin-film deposition) that depend on a careful selection of fabrication parameters can benefit from the ML methods, which can generate optimum results in a time- and cost-effective fashion. Here, such an ML-based optimization approach was applied to a technologically very relevant problem: the encapsulation of copper against chemical attacks from a harsh environment.

The surface passivation of copper is often used in corrosion protection in various applications such as microelectronics, photovoltaics, heat exchangers, gate electrodes, and interconnects.^5−9^ The deposition of thin films is one of the most preferred methods for applying protective coatings.^10−12^ The main challenge for depositing thin films for corrosion protection is eliminating the defects, such as pinholes and structural defects that allow the transport of reactive species in films.^13−16^ Among all the available deposition techniques, atomic layer deposition (ALD) stands out owing to its high-quality thin films with excellent uniformity and conformity. ALD is also often cited for having lower defect densities.^17−21^ However, the defect density on which the corrosion-protection performance depends has to be strictly controlled and minimized for wide-scale applications.^22^ Microparticles, heterogeneities on the substrate, or the presence of an interfacial oxide layer between the substrate and the passivation layer serve as nucleation sites for defects.^23−25^ Also, an inaccurate tuning of ALD process parameters may lead to defects due to undesired surface reactions.^26,27^ Thus, both intrinsic and extrinsic defects can trigger and accelerate the corrosion process locally.^23,27−29^

The performance of ALD thin films for corrosion protection based on the defect density has been addressed by multiple research groups.^8,10,13−16,22−28,30−45^ Among these, the most extensive research is performed on Al_2_O_3_ layers, thanks to their excellent adhesion to many metal surfaces.^38,46^ Zhang et al. showed that the defect density of ALD-Al_2_O_3_ layers on copper determined by copper electroplating was reduced by orders of magnitude (from ∼1.2×10^5^ cm^–2^ to ∼90 cm^–2^) with appropriate substrate surface treatment for efficient nucleation of ALD-Al_2_O_3_.^15^ Vanhaberbeke et al*.* studied the porosity of 20 nm thick ALD-Al_2_O_3_ layers on copper. They reported the number of pinholes as 50 cm^–2^ to 100 cm^–2^ by copper electroplating and the porosity as 8.04 × 10^–2^% to 3.23 × 10^–3^% by linear sweep voltammetry (LSV).^34^ In our previous study performed on copper substrates, we also investigated the corrosion protection of Al_2_O_3_/TiO_2_ and Al_2_O_3_/SiO_2_ bilayers at elevated temperatures.^47^ Even though the ALD layers showed excellent corrosion protection at elevated temperatures, the detailed spectromacroscopic study revealed copper on the surface of the ALD layer. The transport of copper was attributed to the defect structure of the layers, which created the pathway through the film. Some other studies investigated the ALD process parameter effect on the defect density.^28,32,33^ Chang et al. reported that the intrinsic defects nucleate on residual OH ligands originating from the incomplete reactions in the first ALD cycles because of the substrate surface properties.^13^ Even though several studies in the literature have focused on the defect structure of ALD-Al_2_O_3_ and substrate surface properties,^15,22,27^ systematic optimization involving substrate surface properties and ALD process parameters has not been reported. These studies reveal that the correct identification and the tuning of principal parameters are essential in improving the design of experiments and achieving the best corrosion protection with ALD thin films. However, these parameters exist in a continuous and high-dimensional search space, which may require an impractical number of experiments to identify and optimize using manual tuning or exhaustive search methods. In addition, ALD is a relatively slow deposition method, which further complicates manual optimization approaches. That is why alternative methods would be desirable for achieving higher efficiency in terms of experimentation.

Various methods can be applied to multiparameter optimization problems, such as ALD thin film fabrication. Optimization methods based on mathematical models are proposed to reduce the number of experiments while identifying the principal parameters that define a process.^48−50^ One of the most widely used methods is the statistical design of experiment (DOE), aiming at obtaining the most extensive possible information about the produced results with the smallest possible number of experiments.^48,49^ The two-level fractional factorial design (FFD) is used to screen the designs to identify the primary factors that control a process.^49,51^ For the linear regression model, higher than first-order equations are used to identify both individual effects and the interaction effect of input parameters on the results.^48,51,52^ The response surface methodology or the Taguchi method is used to build a model for the relation between input parameters and output results.^48,53−56^ This established model can be used for optimization purposes.^48,55,56^ However, it could be necessary to extend the parameter space repeatedly to build an acceptable model and perform optimization if the mathematical model is not verified by the defined DOE design.^50,57^ Therefore, DOE methods do not explain the relationship between input parameters and output results in all application scenarios. In some cases, they might require more trials to discover proper parameter space to make them applicable and explain relations between input parameters and output results.

There are emerging examples of applying ML methods to optimizing selected parameters using a small number of experiments.^50,58−62^ Among these, the Bayesian optimization (BO) method can employ a probabilistic model (i.e., typically Gaussian processes (GPs)^63^) to represent an unknown function. It sequentially updates this representation by suggesting and testing new data points in that function’s parameter space.^64,65^ BO can be configured to explore the unknown regions in the parameter space of this target function or focus on a specific region of interest in the case of optimization tasks. Therefore, BO has significant advantages compared to optimization methods that require predefined models of the target processes. The ability to operate by testing a small set of data points and using a probabilistic model that can approximate unknown inherent variances in a physical process render BO an attractive method for optimization problems in materials science.^50,66−70^ For example, Yuan et al. studied the optimization of Pb-free BaTiO_3_-based piezoelectric with large electrostrain.^71^ It has also been applied for the optimization of metal oxide’ grain boundary structures for efficient identification,^72^ optimization of high-quality nanofibers,^73^ optimizations of both the alloy composition and associated heat treatment process for the performance of Al-7xxx series alloys,^74^ optimization of low-hysteresis Ni-Ti-based shape memory alloys BaTiO_3_-based piezoelectric with large electrostrains,^57^ and optimization of nanostructures for optimal phonon transport.^75^ Although BO is increasingly used in various fields related to materials science, its applications to the optimization of ALD film properties have not been reported.

In this study, we used the ALD process to deposit Al_2_O_3_ thin films for copper corrosion protection. We proposed a hybrid approach to improve the experimental design process by identifying the principal parameters and optimizing these specified parameters to minimize the ALD films’ defect density for maximum corrosion-protection performance. In our hybrid approach, we first employed a two-level FFD method to rapidly identify the main parameters that affect the defect density. Then, we utilized BO to find the optimum values for the selected process parameters, which is reported for the first time for rapid and adaptive optimization of the defect density of ALD-Al_2_O_3_ films. We studied the properties of the fabricated thin films using wet-chemical etching, X-ray photoelectron spectroscopy (XPS), scanning electron microscopy (SEM), focused ion beam (FIB), and LSV methods. The experimental results showed that the optimization of the identified parameters substantially improved our films’ protective properties against corrosion under harsh conditions. We believe that this hybrid approach will have wide-ranging implications for optimization work in materials science.

## Results and Discussion

### ALD Film Growth

**Figure 1:**
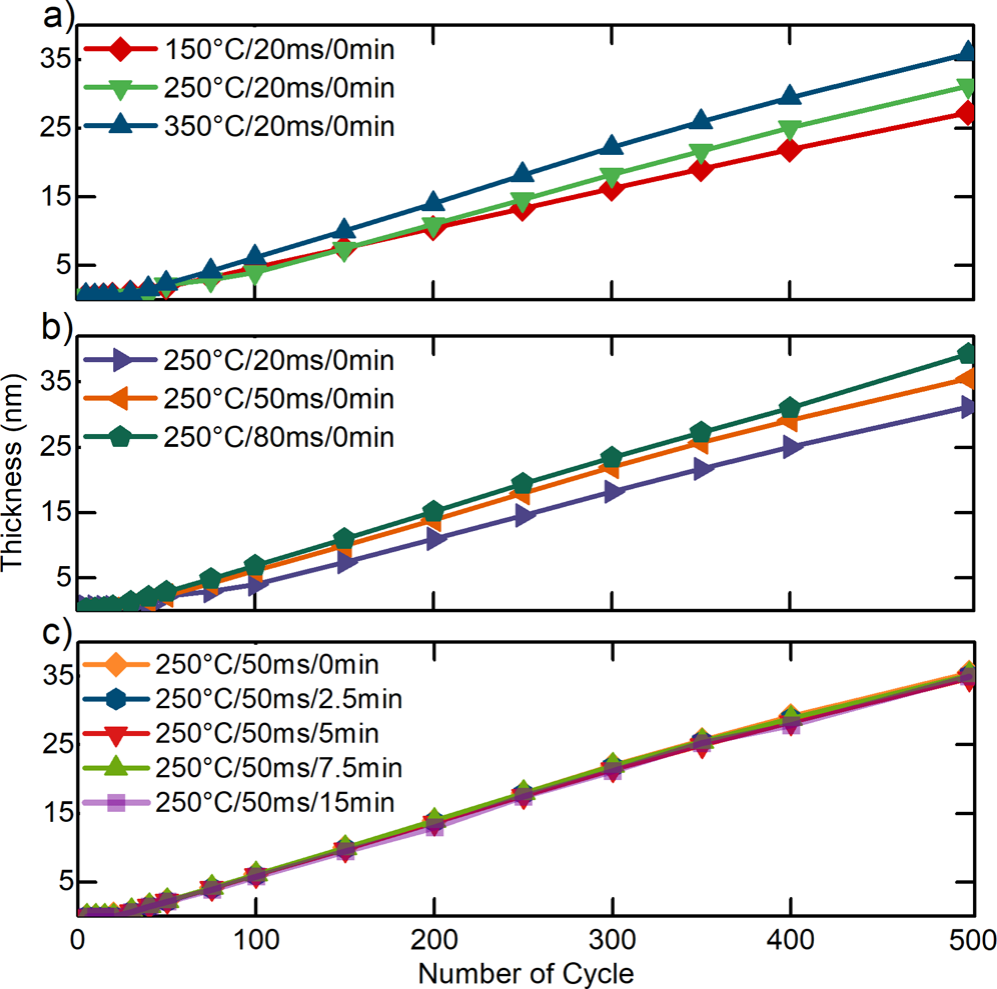
Thickness of the Al_2_O_3_ thin films as a function of the number of ALD cycles for different (a) deposition temperatures, (b) pulse times, and (c) Ar/H_2_ plasma pretreatment. One parameter was varied, while the other two were chosen to be constant for each subfigure.

The Al_2_O_3_ films were deposited for 500 cycles using different process conditions defined in the following section. The film thickness and growth properties were monitored by in situ spectroscopic ellipsometry (SE) measurements. Independent from the process parameters, all Al_2_O_3_ films showed a substrate-inhibited growth mode during the first ALD reaction cycles. ALD depositions showed a linear increase in thickness with the number of cycles. The final thicknesses at 500 cycles were measured between 27 and 40 nm because the growth per cycle (GPC) was changed according to the process parameters. Figure 1a–c shows the growth behavior of Al_2_O_3_ thin films for different values of the deposition temperature, pulse time, and Ar/H_2_ plasma pretreatment conditions, respectively. One of the parameters was varied in each part (Figure 1), while the other two were kept constant. The GPC increased with increasing temperature and pulse time, with a maximum of 0.08 nm/cycle. As indicated in Figure 1c, the GPC remained the same with different Ar/H_2_ plasma pretreatment durations.

According to the GI-XRD analysis, Al_2_O_3_ films were amorphous at all deposition temperatures, as shown in Figure S1, Supporting Information. From the XPS survey spectra, the stoichiometry ([Al]:[O]) ratio was found to be 0.6, which indicates the Al_2_O_3_ composition (Figure S2, Supporting Information). The surface properties of Al_2_O_3_ films according to SEM and optical microscopy are given in Figure S3, Supporting Information.

### Copper Wet Etching

The ALD-Al_2_O_3_-protected copper samples were etched in a nitric acid (40 vol %) solution at room temperature for a duration between 1 and 30 min (Figure S4, Supporting Information). The etching process, which was monitored by optical microscopy, started with small punctiform dots that indicate defects. The number of dots and the dimensions of etched areas were related to the etching duration. An increase in the etching duration also increased the etched area and overlapped the etched areas. It was assumed that longer exposure to acid allowed all kinds of defects such as line defects, sealed line defects, vacancies, and pinholes to be revealed.^34^ Therefore, considering our applications and optimization process, the study here focused on longer acid exposure durations, namely, 30 min, to reveal all defect types. At these long etching durations, the individual etched regions starting off of a single defect started to coalesce. Therefore, counting individual pinholes for longer etching processes was not practical. In this study, we defined the total etched area percentage of copper as an indicator of the initial defect density and the quality of deposition in terms of corrosion protection.

### Reference Al_2_O_3_ Sample

**Figure 2:**
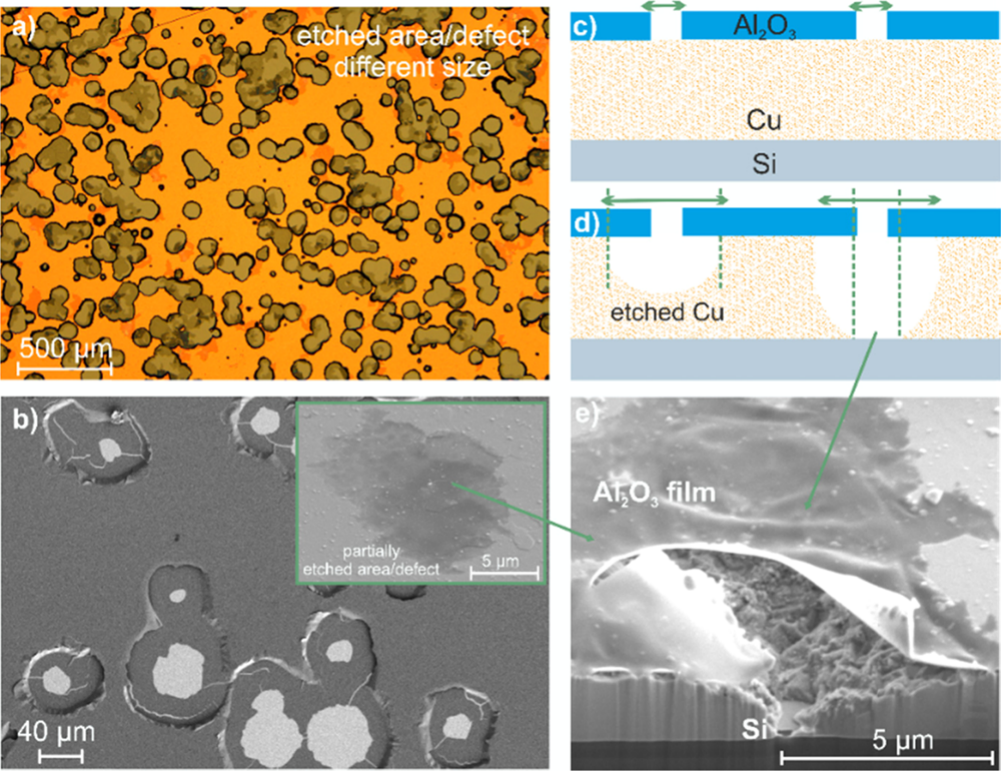
Etching (30 min) of Al_2_O_3_-protected Cu. (a) Optical microscope images from the middle of the area (according to three different samples and five different regions on each sample, 45% of the total area was etched). (b) SEM images from the larger vias. (c) and (d) Proposed sketch for major steps for wet etching, in which the etchant reached the copper surface through the defect, and copper was dissolved below the ALD film. (e) Cross-section images of the presented area that focuses on smaller vias. The standard deposition parameters were used for the reference Al_2_O_3_ film.

The reference Al_2_O_3_ films, before the optimization described in this work, were deposited at 150 °C with 20 ms pulse time and 1980 ms purge time for both precursors TMA and H_2_O without any plasma pretreatment.^35,36,76^ ALD deposition using these standard process parameters was referred to as “reference samples” in the following. These samples were exposed to the nitric acid (40 vol %) etching solution for 30 min. After the acid/water solution reached the copper surface through the defects, the etching reaction took place and generated gaseous and soluble byproducts.^77^ According to Zang et al*.*, the etchant removed copper at the same rate in all directions during the etching process and produced a hemispherical trench.^78^ After 30 min of etching in nitric acid (40 vol %) solution, the typical etched pattern was obtained, which is shown in Figure 2. While Figure 2a,b shows the optical microscope and SEM images, Figure 2c,d shows the proposed sketch of major steps for the wet etching process. The cross-section of a single etch pit is given in Figure 2e, which is in good agreement with the proposed sketch of the trench formation originating from a point-like defect in the ALD layer such as pinholes and progressing until copper is completely removed.^34^ The remaining free-standing film was usually torn in the course of stress relaxation.

The reference sample deposition and wet etching process were repeated three times to determine the batch-to-batch deviation. Additionally, three samples were deposited for each batch to evaluate sample-to-sample deviation (Figure S5, Supporting Information). While the average etched area from three samples was found to be 39.35 ± 10.51% for batch 1, they were 17.54 ± 2.02 and 77.92 ± 13.62% for batch 2 and 3, respectively. The overall mean etched area for three batches was then 44.94 ± 27.32%, calculated by averaging the values with error propagation analysis. The substantial deviation of the etched area percentage and the lack of reproducibility from sample to sample and from batch to batch were attributed to an unoptimized process that leads to an undesired behavior. The significant differences between the samples were a sign that the defects were randomly affected by all steps and not controllable in the complex system as the deposition parameters were not optimized.

### Applications of the FFD

A total of six parameters: O_2_ plasma pretreatment, Ar/H_2_ plasma pretreatment, process temperature, pulse time, atomic layer annealing (ALA), and pressure were screened to identify the key parameters that have a main effect on the defect density for maximum corrosion-protection performance. While Ar/H_2_ plasma and O_2_ plasma pretreatments were applied before the ALD deposition, pulse time, ALA, and pressure were changed during deposition. According to Shih et al., the ALA treatment was defined layer-by-layer as in situ plasma treatment between each ALD cycle.^79^

With a two-level FFD, eight experimental runs were performed (see Methods).^49,80,81^ The high and low levels (two-level) for each parameter were determined in reference to the original deposition parameters (reference sample), which was called “Run 0.” According to the experimental design, O_2_ plasma was applied for 0 and 5 min, and the Ar/H_2_ plasma was for 0 and 15 min. The temperature was set to 150 and 350 °C, and pulse time for 20 and 80 ms. The ALA was defined to 0 and 40 ms, and pressure to 6 and 20 Pa. The etched area percentage was used as the output response. The experimental matrix, which was defined by two-level FFD, corresponding responses, and the main contribution effect of parameters are given in Table 1. The response of each experimental run was calculated for three samples and used as the mean etched area percentage after 30 min wet etching. For each parameter, the contribution (Table 1) was calculated as the ratio of its sum of the square over the total sum of squares.^51,82^ The main effect plot is given in Figure S6, Supporting Information. The optical microscopy images of the Al_2_O_3_ films after wet etching are presented in Figure S7, Supporting Information.

**Table 1:** Process Variables Based on the Two-Level FFD with the Final Film Thickness, GPC, and Etched Area Percentages^a^

	pretreatment		ALD process parameters				thickness (nm)	GPC (nm/cycle)	etched area (%)
	Ar-H_2_ plasma (min)	O_2_ plasma (min)	temperature (°C)	pressure (Pa)	pulse time (ms)	ALA (ms)			
run 0	0	0	150	6	20	0	27.3	0.05	45.00%
run 1	15	5	350	20	80	40	51.2	0.103	47.00%
run 2	0	5	350	6	80	0	44.2	0.075	13.63%
run 3	15	0	350	20	20	0	48.4	0.092	0.03%
run 4	0	0	150	20	80	0	46.1	0.095	1.03%
run 5	0	0	350	6	20	40	54.2	0.103	0.08%
run 6	0	5	150	20	20	40	56.1	0.106	1.77%
run 7	15	0	150	6	80	40	56.7	0.107	0.29%
run 8	15	5	150	6	20	0	26.7	0.048	30.70%
effect	15.80	23.32	7.13	1.32	7.42	1.30			
SS	499.41	1087.8	101.55	3.46	110.24	3.40			
**contribution**	**21.65%**	**47.17%**	**4.40%**	**0.15%**	**4.78%**	**0.15%**			

a The etched area percentage was defined as the response of the system. The contribution to the response (etched area) was calculated and given for each parameter

According to Table 1, the O_2_ plasma had the highest contribution, calculated as 47.17%, followed by the Ar/H_2_ plasma with 21.65%. The deposition temperature and pulse time contributions were found to be relatively high and had approximately the same magnitude of effect on the etched area with 4.40 and 4.78%, respectively. The effects of the ALA and pressure were observed to be even lower with only 0.15% contribution.

Based on the two-level FFD results, the parameters of the ALA and pressure were not considered for the following optimization step because of their low influence on the corrosion-protection performance. According to Shih et al*.*, the ALA process was applied to enhance adatom migration and remove the ligands by energy transformation from Ar plasma.^79^ However, the ALA process showed no improvement in the protection performance of Al_2_O_3_ films based on two-level FFD analysis. On the other hand, even though the O_2_ plasma pretreatment had the highest contribution based on the FFD results, the etched area percentage dramatically increased when the O_2_ plasma-pretreated samples were immersed in the acid solution for 30 min. This negative impact of the O_2_ plasma pretreatment was attributed to the formation of a Cu_2_O film on copper (The thickness of the Cu_2_O film was measured up to 300 nm by in situ SE, and surface properties were analyzed by XPS, as shown in Figure S8, Supporting Information). In conclusion, the O_2_ plasma pretreatment was entirely omitted from the optimization step because of its adverse effect on the protection properties of the deposited Al_2_O_3_ film.

The remaining parameters, Ar/H_2_ plasma pretreatment, temperature, and pulse time, were selected as the influencing parameters on ALD deposition quality. Hence, the two-level FFD analysis allowed us to reduce the number of parameters from 6 to 3 to be considered for the BO step.

### Applications of the BO Design

To minimize the etched area percentage, the Al_2_O_3_ films were deposited iteratively using the process parameter values suggested using the BO method (see Methods). Their values were varied until the etched area percentage reached the target value of 0.03%, which was selected based on the best-performing run (i.e*.*, run 3 in Table 1) achieved during the FFD analysis. The duration of Ar/H_2_ was defined between 0 and 15 min, the deposition temperature, *T*, varied between *T*_min_ = 150 ^°^C and *T*_max_ = 350 ^°^C, and the pulse time *t*_pulse_ was limited to [20–80 ms]. The step sizes were set to 2.5 min and 50 °C for Ar/H_2_ and *T*, respectively. The parameter set for *t*_pulse_ was defined as {20,40,50,60,80} ms using a nonfixed step size. The range of values for the investigated parameters with the given step sizes yielded a total number of 125 distinct parameter sets in Θ in our experiments.

The hyperparameters for the GP and BO were listed as the prior mean μ(θ), noise in the collected data *σ*_n_^2^, signal variance *σ*_f_^2^, length scale *l*_c_ for each dimension of the parameter space R^d_c_^, and exploration-exploitation tradeoff constant ξ. Considering the etched area could have a value in the range of [0–100%], μ(θ) and *σ*_f_ were selected as 50% and 25%, respectively, to contain the actual defect density function inside the 95% confidence interval of the prior. The standard deviation of the noise in the collected data *σ*_n_ was set to 0.01%, selected based on the two-level FFD experimental results near the optimization goal. The length scales were set to one-fourth of the total range of each corresponding parameter.^83^ Lastly, *ξ* was chosen as 0.9 to balance the exploration-exploitation tradeoff.

**Figure 3:**
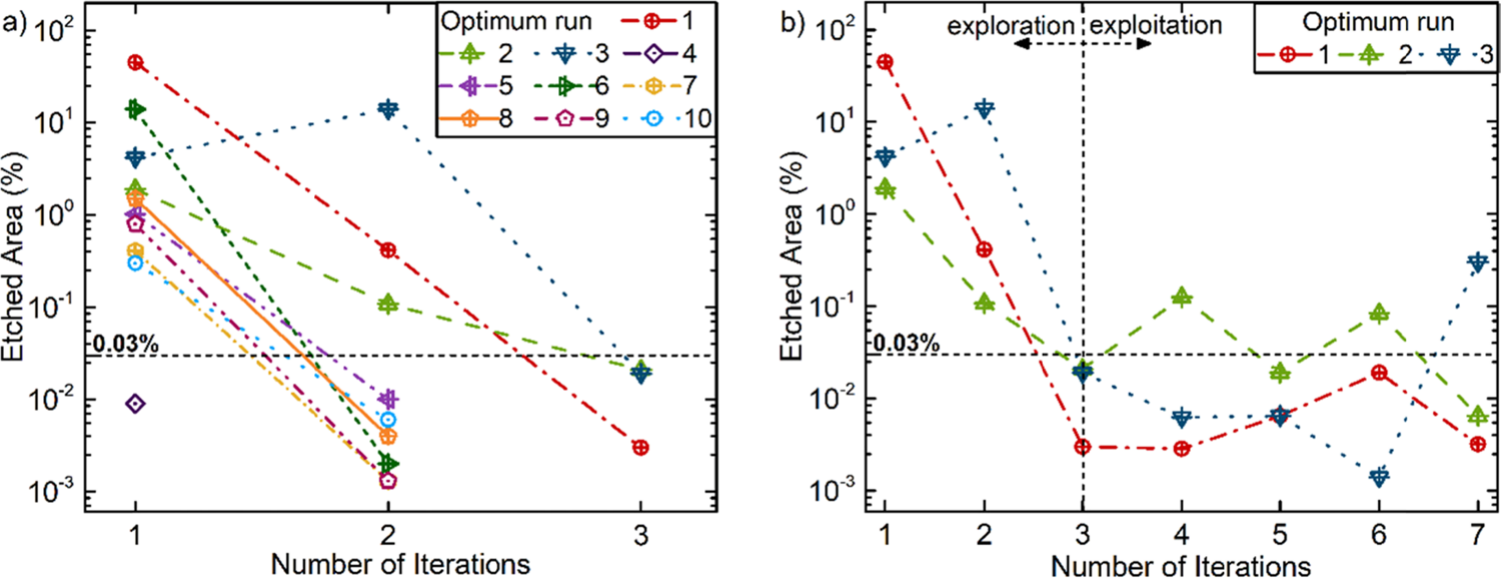
BO process showing the minimization of the etched area percentage for each iteration. (a) 10 parallel optimization runs with exploration orientation until the etched area percentage reached the target value, 0.03%, pointing to a significant decrease in the defect density. (b) Following experiments with exploitation orientation with three parallel optimization runs to seek better performances for lower defect density in the system.

The optimization was started with 10 parallel runs of independently selected sets of parameters, seven of which were randomly chosen and the remaining with known-outcome conditions from previous experiments. In these optimization runs, the expected improvement (EI) was selected as the acquisition function. Each optimization run was stopped when the target threshold, 0.03%, was achieved in the etched area percentage. Table 2 summarizes the results of experiments for these 10 independent optimization runs. The table additionally lists the tested parameter sets, corresponding etched area percentages, and selected optical microscope images for low and high defect densities. Figure 3a shows the obtained etched area percentage values at each step of the optimization runs. All of the optimization runs reached the target threshold in the second or third iteration of the optimization process, which underlines the success of this optimization approach in minimizing the total number of experiments.

**Table 2:**
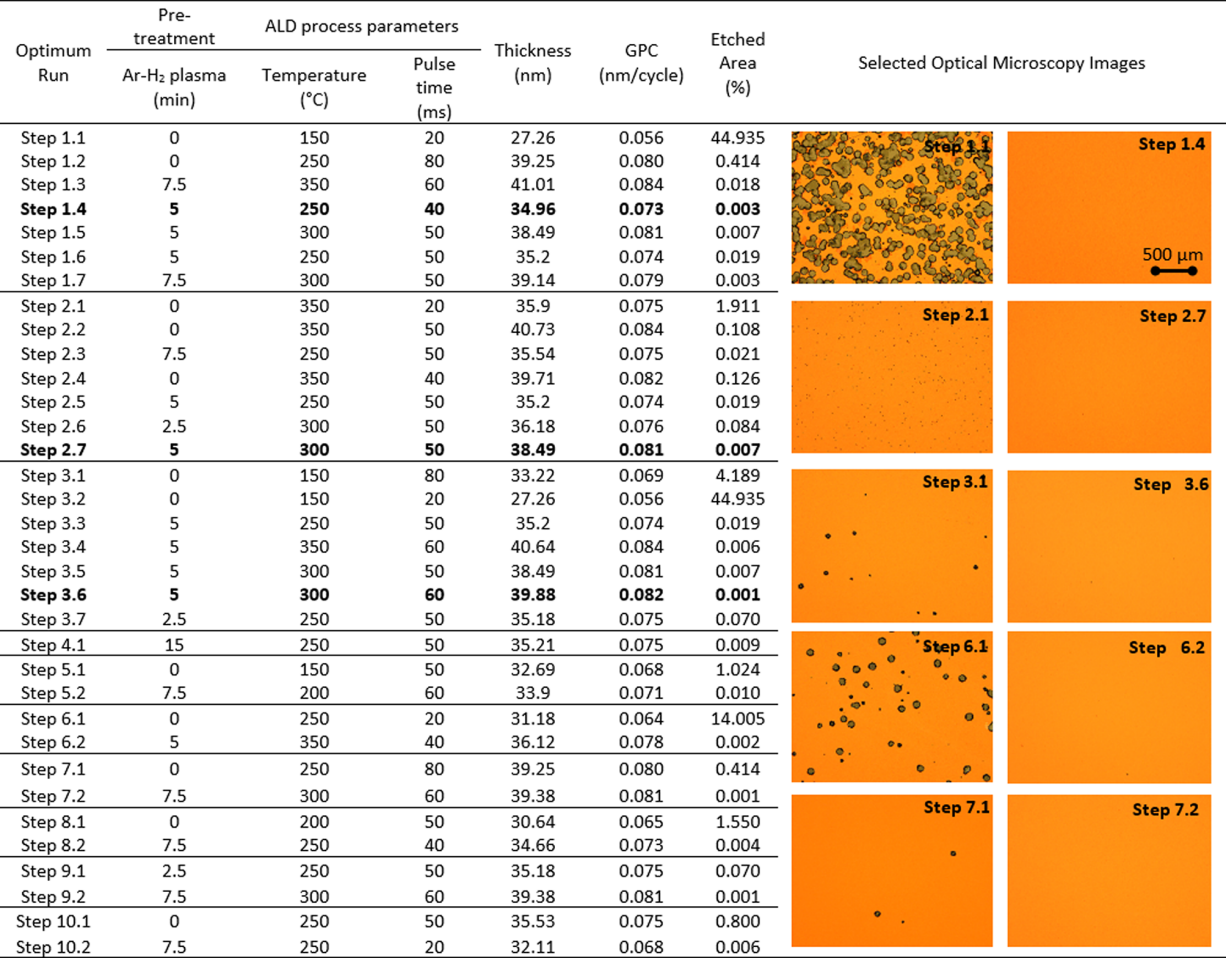
Experimental Parameters and Results of 10 Parallel Optimization Run for Achieving the Defect Density Target^a^

a Each optimization step is labeled by the identifier of the optimization run and the corresponding iteration of the BO (e.g., Step 1.2). The table shows the experimental parameters at each step, the final ALD thickness of the Al_2_O_3_ thin film, the growth rate GPC, and the etched area percentage. The selected optical microscope images are given for the best and worst observed etched area percentages. Step 1.1 shows the reference sample

To continually search the optimal state through the iterative process and gain more information about the system, additional experiments have been performed on the first three optimization runs (i.e., shown as optimum run 1–3 in Figure 3a). To prioritize exploitation, the probability of improvement (PI) was chosen as the acquisition function in BO throughout these additional experiments. Within the subsequent four iterations of the first optimization run, the etched area percentage improved in the first iteration. However, the second and third optimization runs achieved the lowest defect densities in the following iterations. Figure 3b illustrates these three optimization runs, including the exploration-oriented steps (first three iterations) and exploitation-oriented follow-up steps (subsequent four iterations) of the BO.

The presented results demonstrate the applicability of BO for the optimization of the ALD thin film fabrication process with the given objective function. At least four orders of magnitude improved the etched area percentage of the optimized Al_2_O_3_ layers compared to the reference state. The optimal parameters were found in less than three iteration steps out of the complete set of 125 parameter triplets. The reliability of the optimal parameters was evaluated by repeating the procedure shown in the optimal run 3.6. The Al_2_O_3_ deposition was repeated three times under the same conditions for three samples for each deposition (Figure S9 Supporting Information). The mean etched area was found to be 0.0027 ± 0.0012, 0.0032 ± 0.0003, and 0.0037 ± 0.0017% for the three batches, respectively, and it was 0.0014 ± 0.0007% for the optimal run 3.6. Compared to the reference Al_2_O_3_ sample, the mean etched area percentage was decreased from 45 ± 27 to 0.003 ± 0.001% with the optimization of process parameters (Table S1, Supporting Information). While some batch-to-batch differences were still observed, the overall etched area remained well below the target value of 0.03%, demonstrating the successful application of the BO approach.

### Effect of the Ar/H_2_ Plasma Pretreatment on the Defect Density

**Figure 4:**
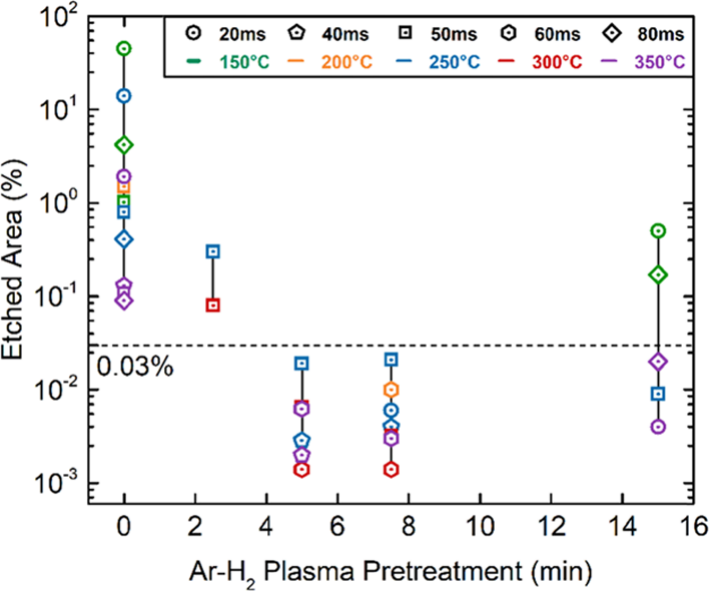
Effect of the Ar/H_2_ plasma pretreatment on the etched area percentage (defect density) of Al_2_O_3_ thin films. The data were collected based on the plasma pretreatment time at different deposition temperatures and pulse time to highlight the main effect of the plasma pretreatment. The pulse time steps are represented by different symbols and the temperature by color codes. The 0.03% etched area was labeled as the optimization target of BO.

The Ar/H_2_ plasma pretreatment influence on the corrosion-protection properties of Al_2_O_3_ was studied with a remote plasma configuration inside an ALD chamber. The pretreatment time was varied from 0 to 15 min with 2.5, 5, and 7.5 min interval steps. After the pretreatment, the ALD process was immediately started for 500 cycles of Al_2_O_3_ deposition. The plasma pretreatment effect on the etched area percentage, given in Figure 4, is a projection of four-dimensional experimental data into a two-dimensional space. The experimental data are collected from the BO runs (Table 2) and from the data obtained by four additional experiments collected during parameter limit search (Table 2, Supporting Information).

The resulting etched area percentage was found to be between 45 and 0.09% without plasma pretreatment (0 min) and decreased to a minimum of 0.07% with the application of 2.5 min plasma pretreatment. However, both cases could not reach the optimization target value of 0.03%. The defect density was improved with increasing plasma pulse time from 2.5 min to 5 min and reached the target value for a range of different ALD process parameters. The lowest etched area percentage was found to be 0.001% with 5 min plasma pretreatment. For a longer duration of plasma pretreatment, similar etched area percentage values (below 0.03%) were obtained except for the two experiments deposited at 150 °C. Longer exposure to plasma species did not further improve the corrosion-protection performance of the Al_2_O_3_ film. Therefore, the plasma pretreatment duration was found to be optimum at around 5 min of application. These results indicate that the plasma pretreatment had the most significant effect on defect density, which agrees with two-level FFD results. Despite the significant improvement of the defect density with the plasma pretreatment, the results of the 15 min pretreatment showed that it is still not enough to reach the defect density goal below the target value on its own.

The effect of the plasma pretreatment was attributed to the difference in interface between Cu and Al_2_O_3_ film. At first, Cu samples were cleaned using acetic acid to remove the native oxide and residuals from the copper surface. The Cu_2_O thickness after this cleaning process was measured to be 2.5 nm by in situ SE before the plasma pretreatment. This thin oxide layer was attributed to the formation of native oxide during a few minutes of the sample transfer and the installation into the ALD chamber, according to work by Chavez et al*.*^84^ The Al_2_O_3_ films would be deposited on this air-formed oxide layer if there was no plasma pretreatment application in the designed experiment. By applying the plasma pretreatment, the air-formed oxide layer is removed according to the in situ SE data (Figure S10, Supporting Information). However, according to Mirhashemihaghighi et al*.*, the Cu_2_O layer can regrow on copper because of water vapor exposure until full coverage of the surface by the Al_2_O_3_ film.^14,25^ Inside the ALD chamber, the well-controlled conditions lead to a homogenously regrown Cu_2_O layer. Therefore, the improvement of the corrosion-protection properties with the plasma pretreatment was attributed to the homogenously grown, clean copper oxide layer rather than the air-formed native oxide, which was in good agreement with the study by Harkönen et al.^32^ Moreover, homogenously grown oxide layers lead to decreased channel defects and hence significantly improve the corrosion-protection properties, according to Mirhashemihaghighi et al*.*^24^ While wet etching is often used to remove the native copper oxide layer before ALD deposition,^25^ our results show that an in situ Ar/H_2_ pretreatment was additionally necessary to control the interface and significantly improve the quality of the corrosion protection of copper by ALD-Al_2_O_3_ films.

### Effect of ALD Process Parameters on the Defect Density

**Figure 5:**
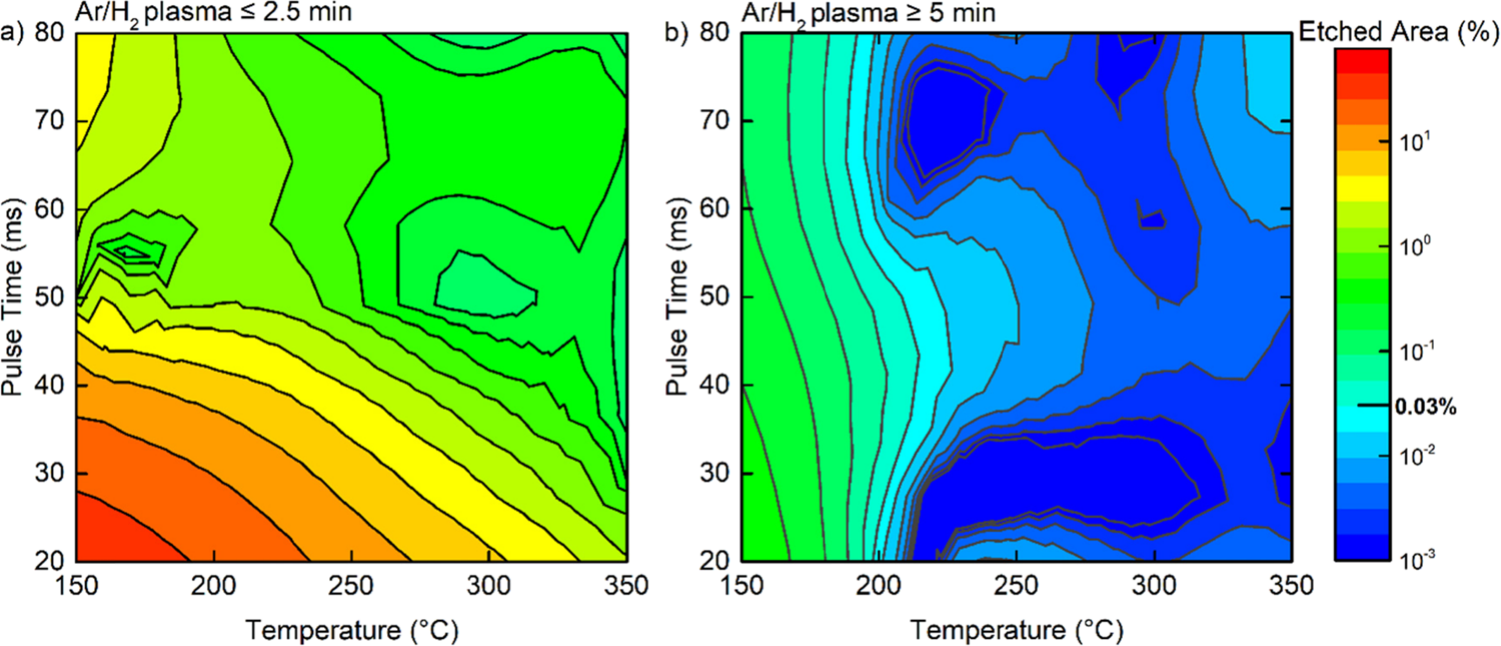
Effect of temperature and pulse time on the defect density. The data were organized based on temperature and pulse time for (a) shorter (≤ 2.5 min) and (b) longer (≥ 5 min) plasma pretreatment after interpolation. The pulse times were indicated on the *y*-axis and temperature on the *x*-axis. The 0.03% etched area was labeled on the color scale as the optimization target of BO. The etched area percentages are shown on the same color scale.

The influence of the deposition temperature and pulse time on the corrosion-protection properties of Al_2_O_3_ is discussed in two parts. While the first part focuses on the correlation between temperature and pulse time without the effect of plasma pretreatment (≤ 2.5 min), the second part focuses on the combination of temperature and pulse time with plasma pretreatment (≥ 5 min). They are illustrated in Figure 5a,b, respectively. The temperature was varied between 150 and 300 °C, and the pulse time, between 20 and 80 ms.

The lowest temperature (150 °C) and the shortest pulse time (20 ms) result in 45% of the copper film being etched during acid immersion. On the other hand, the highest temperature (350 °C) and the longest pulse time (80 ms) resulted in the etching of 0.09% of the total copper area. Therefore, higher temperature and longer pulse time were found to be beneficial for reducing the defect density when the plasma pretreatment was less than or equal to 2.5 min (Figure 5a). When the plasma pretreatment was more than or equal to 5 min, the optimization target of 0.03% was achieved for all pulse times as long as the deposition temperature was above 200 °C (Figure 5b). Among the two parameters, the temperature was therefore identified to have a more significant influence than the pulse time on the corrosion-protection properties of Al_2_O_3_ thin films.

The effect of temperature on the defect density can be attributed to the type of reactive sites and the effect of temperature on the preferred reaction mechanism.^85^ The reactive surface sites affect the amount and the type of chemisorbed species. A lower defect density with increased temperature may be related to the higher reactivity of the species. Furthermore, higher temperatures do likely provide sufficient thermal energy to drive new reactions that do not occur at lower temperatures.^86^

The optimized conditions determined by the BO indicate that copper samples should be treated with the Ar/H_2_ plasma for 5 min. While shorter plasma pretreatment was not enough, the longer pretreatment did not significantly improve the defect density and could even be detrimental. The deposition temperature should be above 200 or 250 °C for even better results. For Ar/H_2_ plasma above 5 min, the pulse time’s impact on defect density was less significant overall. Even with the shortest pulse time (20 ms), the target value of 0.03% could be achieved. In general, a shorter pulse time is advantageous and desired as it reduces the deposition time, conserves precursors, and hence reduces the associated production costs. The layer thickness was not significantly crucial for the defect density, as shown in Figure 1 and Table 2. While the thickest layers (40 nm) were obtained at 350 °C, the optimized defect density (<0.03%) was achieved even in 32 nm thick Al_2_O_3_ layers with plasma pretreatment at 250 °C. Figure S11, Supporting Information, represents the relation between the deposited thickness and etched area percentages.

### Electrochemical Corrosion-Protection Properties of Optimized Layers

**Figure 6:**
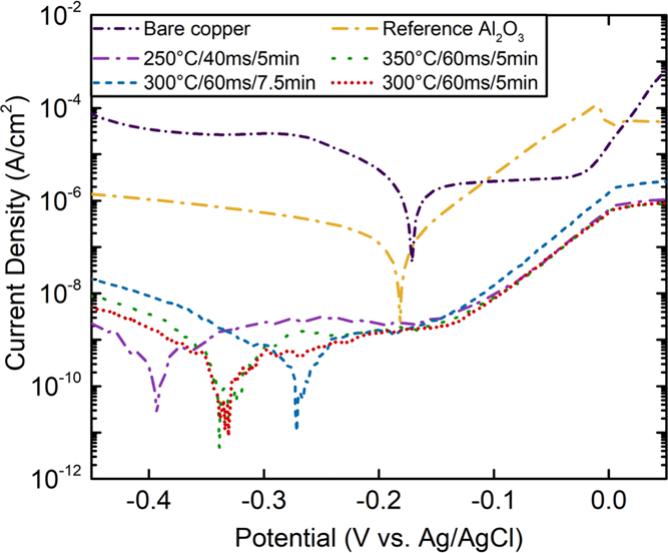
Polarization curves of bare copper, reference Al_2_O_3_ film, and selected optimized Al_2_O_3_ films.

Finally, we studied the electrochemical response of the optimized layers using LSV. The polarization curves for bare copper, the reference Al_2_O_3_ layer, and selected optimized-Al_2_O_3_ layers are shown in Figure 6. The polarization curves, analyzed using the Tafel fit, were used to calculate the corrosion current, polarization resistance, and electrochemical porosity.^25,87,88^ The calculated values are given in Table 3. The corrosion potential of bare copper shifts toward negative values with Al_2_O_3_ deposition, indicating improved corrosion resistance. The corrosion current density was found to be 1.5 × 10^–5^ A cm^–2^ for bare Cu and dropped to 9.9 × 10^–7^ A cm^–2^ with the presence of reference Al_2_O_3_ films. While the presence of reference ALD-Al_2_O_3_ film leads to a general decrease in the current density, the selected optimized Al_2_O_3_ films show a marked reduction in the corrosion current density with approximately five orders of magnitude compared to bare Cu. Even though different process parameters were used for the deposition of the optimized Al_2_O_3_ films, similarly reduced corrosion current densities were obtained between 3.2 × 10^–10^ and 9.0 × 10^–11^ A cm^–2^, which indicates the successful optimization process by BO.

**Table 3:** Corrosion Current (*I*_corr_), Polarization Resistance (*R*_p_), and Porosity^a^

	sample run	*I*_corr_ (A cm^–2^)	*R*_p_ (Ω)	porosity (%)
bare Cu		1.50 × 10^–5^		
reference Al_2_O_3_	Step 1.1	9.87 ×10^–7^	1.2 × 10^5^	6.58
250 °C/40 ms/5 min	Step 1.4	3.02 × 10^–10^	3.0 × 10^7^	0.02
300 °C/60 ms/5 min	Step 3.6	9.00 × 10^–11^	8.2 × 10^7^	0.0007
350 °C/60 ms/5 min	Step 3.4	1.95 × 10^–10^	5.5 × 10^7^	0.0013
300 °C/60 ms/7.5 min	Step 7.2	2.42 × 10^–10^	4.5 × 10^7^	0.0016

a The polarization curve was used to calculate the values.

The electrochemical porosity for reference Al_2_O_3_ films was found to be 6.58%, according to the calculations defined in the Methods Section. With the optimization of the process parameters, the electrochemical porosity dropped from 6.58% to the values of 0.02 and 0.0007%. Even though the etched area percentages (from wet etching analysis) and porosity (from LSV analysis) showed similar values, the mechanism behind the two measurement techniques was different. The etched areas were formed when the etching solution was in direct contact with the copper surface. In the LSV method, the porosity was defined based on the measured current with contribution from charge transport, Poole-Frenkel effects, and trap-assisted tunneling phenomena.^34^ Therefore, the direct comparison of the results is not possible. However, a positive correlation between the two can be expected.

**Figure 7:**
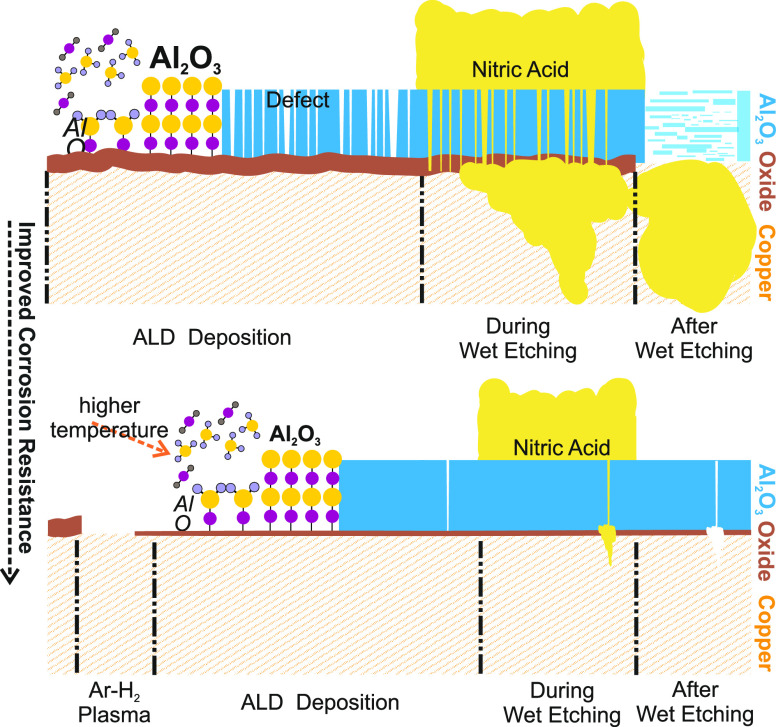
Schematic representation of the corrosion-protection performance of ALD thin films before and after optimization. While the top side of the figure describes the process without optimization, the bottom side describes the improvement. With the optimization process, the applications of plasma cleaning (before ALD deposition) and increased temperature were found to be the most important parameters to achieve higher resistance against corrosion.

The improved corrosion-protection properties of optimized ALD-Al_2_O_3_ films were exhibited using wet etching and LSV techniques. Moreover, the following sketch (Figure 7) illustrates how corrosion-protection performance was improved by process optimization. According to Table 3, similar corrosion current densities and electrochemical porosities were further obtained for process parameters, which indicates once more the necessities of process optimization. In the literature, different corrosion current densities were reported.^31,89,90^ For example, corrosion current densities of 8.3×10^–7^ A cm^–2^ and 3×10^–8^ A cm^–2^ were found for 29 nm thick Al_2_O_3_ films on copper by Daubert et al. and Chai et al., respectively.^36,38^ Indeed, Mirhashemihaghighi et al*.* reported a current density of 8×10^–11^ A cm^–2^ for 50 nm thick Al_2_O_3_ on electropolished and cleaned copper.^8^ We report a current density as low as 9×10^–11^ A cm^–2^ even for 32 nm thick Al_2_O_3_ on copper in the present study.

## Conclusions

The microscopic mechanisms of idealized ALD processes are fairly well understood. However, in practice, the deposition process deviates from this idealistic view. Actual samples deposited in real chambers often exhibit intrabatch and interbatch variability, which can be attributed, among other things, to local variations on sample surfaces or differences in chamber designs, respectively. Therefore, processes usually need to be optimized from scratch when significant changes are made, such as changing the substrate or the deposition chamber. Exhaustive searches to find global optimum can be prohibitively impractical and expensive. The remaining choices are either a theory-guided heuristic search, which can get stuck in a local optimum or a design-of-experiment approach such as the FFD or Taguchi method or some variations. Such methods still require tens of different experiments to generate a response surface.

In this work, we successfully deployed a hybrid BO algorithm to find the best process parameters for depositing Al_2_O_3_ thin films toward reliable encapsulation of the copper metal. As a result, the defect density of the Al_2_O_3_ film was significantly decreased, and the corrosion-protection properties of the layer were considerably improved.

The main process parameters that affect the defect density were identified using a two-level FFD method. Ar/H_2_ plasma pretreatment, temperature, and pulse time were selected from six process parameters according to their higher impact on the corrosion-protection performance. The two-level FFD analysis allowed us to reduce the number of parameters from six to three to be considered for the BO step.

BO was successfully applied for the first time to maximize the corrosion-protection performance of ALD-Al_2_O_3_ thin films. The BO was capable of finding optimal parameters in only a few iterations. The efficiency and efficacy of the BO were demonstrated by repeating the optimization 10 times independently and finding the target value of 0.03% etched area in less than three iterations. A repeatability analysis showed the mean etched area to be 0.003 ± 0.001% for three batches. Compared to the reference Al_2_O_3_ film, the etched area was decreased from 45 ± 27 to 0.003 ± 0.001% by optimizing process parameters. We demonstrated how BO could be efficiently applied to optimize the ALD processes and their parameters.

The Ar/H_2_ plasma pretreatment and temperature were found to impact the corrosion-protection performance significantly. The plasma pretreatment duration of 5 min was enough to increase the corrosion-protection performance of Al_2_O_3_ films significantly. After 2.5 min long plasma pretreatment, a deposition temperature above 200 °C and a pulse time of more than 60 ms were required to reach the optimization target. Once the temperature was increased above 250 °C and the plasma pretreatment was applied for 5 min, the optimization goal was achieved independent of the pulse time.

Compared to a bare copper sample, the optimized layers showed improved corrosion protection by more than five orders of magnitude with a corrosion current density of 9×10^–11^ A cm^–2^, one of the lowest reported in the literature for ALD-Al_2_O_3_ films. The improvement of the current density demonstrated the importance of the optimization process for the corrosion-protection performance.

Here, we demonstrated that BO, an algorithm to find the global optimum for a given objective and cost function, can efficiently reach the target optimization goal for an ALD process with a fair number of parameters in only a few steps. Therefore, this study aimed at finding an efficient optimization route for the desired function of being resistant to harsh environments. Knowing that we can easily transfer ALD processes in between different chambers and onto other substrates with this method, attention can be focused on the stack or device design. Furthermore, this approach will allow us to utilize the insights into this research to further studies where the material stack is more complex or multiple functions have to be fulfilled at the same time.

## Methods

Copper (Cu) samples (99.999% purity) were electrodeposited on p-type Si (100), with ∼1.5 μm Cu thickness, ∼3 nm native oxide, and ∼6 nm surface roughness. Before ALD deposition, all samples were rinsed with acetic acid for 1 min to remove residuals and oxide layers from the Cu surface. The Al_2_O_3_ films were deposited using an ALD system with a plasma source attachment (Sentech GmbH, Germany). For the deposition of Al_2_O_3_, trimethylaluminum (TMA) and ultrapure H_2_O were used as precursors under constant nitrogen flow. The reference 30 nm thick Al_2_O_3_ films (nonoptimized) were deposited with precursor pulse for 20 ms and purge of 1980 ms during 500 cycles at 150 °C. The growth properties and final thickness of Al_2_O_3_ on copper were analyzed using in situ spectroscopic ellipsometry (SE, SENresearch 4.0., Sentech, Germany).

Ar/H_2_ plasma pretreatment, O_2_ plasma pretreatment, deposition temperature, pulse time, ALA, and pressure were selected as possible parameters that influence the defect density of ALD films. While Ar/H_2_ plasma and O_2_ plasma pretreatments were varied before the deposition starts, pulse time, ALA, and pressure were varied during ALD deposition. The plasma pretreatments were applied under 20 Pa, with 200 sccm gas flow and 200 W plasma power. Ar plasma was pulsed in between each ALD cycle for 40 ms with 200 sccm gas flow and 200 W plasma power for the ALA process. The purge time was extended to 5 s for all the processes to ensure the self-limiting ALD growth based on different pulse time parameters. All Al_2_O_3_ layers were deposited for a constant 500 cycles as the GPC is dependent on the process parameters. The final thickness varied accordingly. The resulting thicknesses were reported in the results part. The two-level FFD design was applied to identify the main parameters for maximum corrosion-protection performance rapidly. The BO approach was then implemented to find the optimum values for the selected process parameters.

The Cu-Al_2_O_3_ samples were etched for 30 min in a nitric acid etching solution (40 vol %) at room temperature. While Cu was dissolved immediately in a nitric acid solution, Al_2_O_3_ was found to be resistive.^91^ The Cu-Al_2_O_3_ samples’ etching started with small punctiform dots that increased in size and number over-etching time. Because of the difficulty of identifying individual defects, the defect density was assumed to correlate positively with the total etched area of copper. Therefore, for wet-chemical etching tests, the ratio of the etched area was utilized to the whole area, that is, the etched area percentage, as an indirect indicator of the defect density. The etched areas were analyzed by optical microscopy and Fiji-ImageJ software. Three samples were etched in fresh nitric acid solutions for each experiment. Five different regions were inspected from each sample.

A Gamry corrosion cell was used with a 1 cm^2^ working electrode, platinum mesh counter electrode, and 0.1 M sodium chloride (NaCl) electrolyte to perform LSV. The cell was allowed to reach equilibrium at an open-circuit potential (OCP) before LSV measurements were taken. The LSV data were collected using an Autolab potentiostat (Ecochemie Inc., model PGSTST 302 N) at a rate of 0.1 mV s^–1^ from −0.6 to 0.5 V versus an Ag/AgCl reference electrode. The corrosion potential, corrosion current, polarization resistance, and porosity were calculated from the polarization curves obtained using LSV and Tafel analysis.^87^ The electrochemical porosity (*P*) was calculated according to the methodology developed by Tato et al. (eq 1).^32,88^
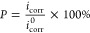
1where *i*_corr_^0^ and *i*_corr_ are the corrosion current densities of the uncoated and coated copper samples, respectively.

### Two-Level FFD

In this study, a two-level FFD was used to select the main parameters (factors) from six factors potentially influencing the ALD deposition quality. The two-level FFD is helpful when used at the beginning of an experimental study to determine more influencing parameters with a few experiments.

A two-level FFD is defined by 2^*k*–*p*^, where *k* is the number of experimental parameters designated as factors, and *p* is the fraction of the factorial design (resolution III, 1/8).^48,80^ To evaluate the main effects of six parameters on the etched area percentage, 1/8 fraction was used to decrease the number of experiments. Instead of a full-factorial design (2^6^), only eight experimental runs were performed in the design of two-level FFD with resolution III (2_III_^6 – 3^).^49,81^ Because of selecting a two-level design, the first-order polynomial equation was used for the linear regression model.

For the design of the experiment (DOE), each independent variable was set as the upper and lower limit, which corresponded to high and low levels in the two-level FFD. The experimental sequence was randomized to minimize systematic errors. The contribution of the parameters was analyzed using the Minitab R17 software.

### GP and BO

This study aimed at minimizing the ALD film’s defect density to improve the corrosion protection of the ALD-protected copper samples. Therefore, the BO was applied on the following cost function, which was defined as
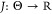
2which mapped the parameter set to a scalar cost value, which was the etched area percentage (as a proxy for the defect density) in our case. Using this definition, the global optimization problem was defined and formulated as
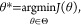
3where Θ denotes the complete search space, θ the process parameters, and *J*(θ) the defect density for a given θ.

Because of the nonlinear response of the ALD process to the parameter changes and the lack of an accurate analytic model of the system, the cost function shown in eq 3 was approximated using the data collected from physical experiments. However, the observed experimental results had inherent uncertainty because of the noise in the measurements and variations during the experiments. To include these uncertainties in the approximated model, to overcome the sparsity in the data, and to make probabilistic predictions for the unobserved design parameter sets, the cost function *J*(θ) was modeled using GPs following a previous study:^83^
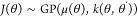
4

Here, the objective cost function *J*(θ) can only be measured with noise, so the experimentally observed defect density *Ĵ*(θ) was defined as
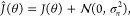
5where  represents the inherited noise in the experimental measurement of the defect density with a Gaussian distribution with zero mean and variance σ_*n*_^2^.

As a nonparametric model, GP was defined by its prior mean μ(θ) and covariance function *k*(θ, θ^’^) that represented the covariance between the data points in θ, that is, parameter sets in our experiments. Here, *k* is the kernel function, which describes this covariance between the data points using the squared exponential method as follows:
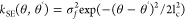
6where *l*_c_ is the length scale that defined the rate of variation in the modeled function for each dimension of the parameter space. In other words, the length scale defined the distance between the two data points where they could significantly influence each other. That is why short-length scales are used to model quickly varying functions, whereas long-length scales are used to model slowly varying functions.^63^ The signal variance σ_*f*_^2^ described the width of distribution, for example, high σ_*f*_^2^ means higher uncertainty in the predictions of the unobserved θ.

BO is a sequential optimization method that updates its model prediction with every step in a finite number of *N* experiments. Throughout a single step of BO, the posterior distribution of this GP model, *J*_post_(θ), is updated based on the observed experimental data D = {θ_*i*_, Ĵ(θ_i_)}_*i* = 1_^*N*^, and then, the defect density for a new θ is predicted using the posterior mean and variance as follows.
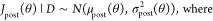
7
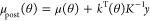
8
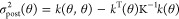
9where *k*(θ) and *y* ∈ *R*^N^ are described as *k*(θ) = *k*(θ, θ_1 : *n*_) and *y* = *Ĵ*(θ_1 : *n*_) – μ(θ_1 : *n*_) for θ_1 : *n*_ ∈ D, and K ∈ R^*N* × *N*^ is the covariance matrix for all observed data points with *K*_i, j_ = *k*(θ_i_, θ_j_) + δ_i, j_σ_*n*_^2^ and i, j ∈ (1,2, ..., N), respectively. δ_i, j_ is the Kronecker delta and σ_*n*_^2^ is the noise in the collected data set.^83^

The BO was utilized throughout the physical experiments to select the following parameter set to be tested, θ_next_, which maximized the acquisition function α_acq_(θ). In this study, two different acquisition functions were used: the PI^92^ and EI.^93^ The PI focuses on the probability of possible cost values below some threshold ξ J*, where J* is the lowest cost function value observed so far, and ξ is the constant controlling the balance between exploration and exploitation.^92^ EI also considers the expected magnitude of such an improvement by considering the variance in the prediction.^92,93^ Therefore, EI was used throughout the first stage of the optimization for exploration and exploitation purposes, and PI was used in the second stage for exploitation only.^94^

A single iteration of the BO with GP was run following the steps given below:

1. BO selected a new parameter set θ maximizing the acquisition function based on the GP model prediction.
2. A physical experiment was conducted with the selected θ, and defect density *Ĵ*(θ) was measured.
3. The GP model was updated with the newly collected data from the experiment for the next iterations of BO.

## Supporting Information Available

ALD-Al_2_O_3_ film properties by GI-XRD, XPS survey scan and SEM/optical microscope images, reference state defect density based on the exposure time and its repeatability, two-level FFD analysis, Cu-Al_2_O_3_ interface differences by XPS for O_2_ plasma pretreatment, repeatability analysis of optimized Al_2_O_3_ films, additional experimental details, in situ SE analysis for Ar/H_2_ plasma pretreatment, and etched area percentage according to thickness (PDF)
