# Supplementary material for: Bayesian Machine Learning for Efficient Minimization of Defects in ALD Passivation Layers
Source: ACS Appl Mater Interfaces. 2021 Nov 4;13(45):54503–15. doi: 10.1021/acsami.1c14586 (PMC8603353; doi:10.1021/acsami.1c14586)
Supplement: Supplementary file 1 — am1c14586_si_001.pdf [file am1c14586_si_001.pdf]

SUPPORTING INFORMATION

Bayesian Machine Learning for Efficient Minimization of Defects in ALD Passivation Layers

Gül Dogan[1,2,*], Sinan O. Demir[2], Rico Gutzler[3], Herbert Gruhn[4], Cem B. Dayan[2], Umut T. Sanli[2], Christian Silber[1], Utku Culha[2,*,†], Metin Sitti[2], Gisela Schütz[2], Corinne Grévent[1], and Kahraman Keskinbora[2,*,††]

[1] Robert Bosch GmbH, Automotive Electronics, Postfach 13 42, 72703 Reutlingen, Germany

[2] Max Planck Institute for Intelligent Systems, Heisenbergstr 3, 70569 Stuttgart, Germany

[3] Max Planck Institute for Solid State Research, Heisenbergstr 1, 70569 Stuttgart, Germany

[4]Robert Bosch GmbH, Corporate Sector Research and Advance Engineering, Robert-Bosch-Campus1, 71272 Stuttgart, Germany

† **Current address:**

Munich School of Robotics and Machine Intelligence, Technical University of Munich, 80797 Munich, Germany

†† **Current address:**

Massachusetts Institute of Technology, 77 Mass Avenue, Cambridge, MA 02139, USA

*Corresponding Authors:

kahraman@mit.edu, utku.culha@tum.de, guel.dogan@de.bosch.com

Keywords: Atomic layer deposition, copper, defect density, wet etching, Bayesian optimization

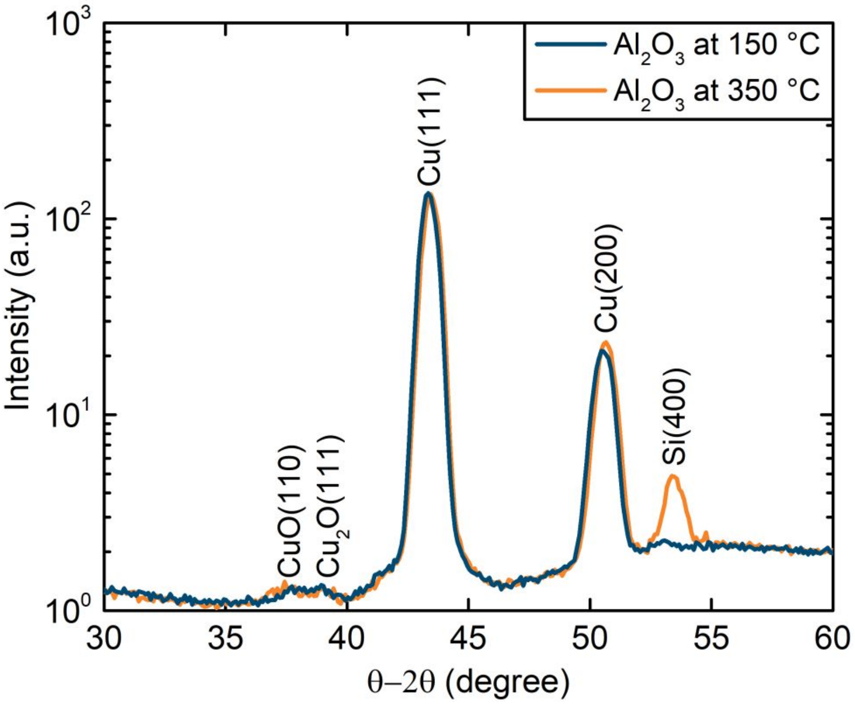

Figure S1. GI-XRD of ALD-Al_2O_3 on copper for lowest and highest deposition temperatures that used in this work. Both diffraction data showed the amorphous phase of Al_2O_3. The Cu (111) and (200) metallic peaks were detected additional of the broad peak at around 38° which was the mixture of copper oxide phase. The Si(400) peak came from the substrate which depends on the relative in-plane orientation of the substrate with respect to the measurement direction. It does not signify a crystallographic change in the structure.

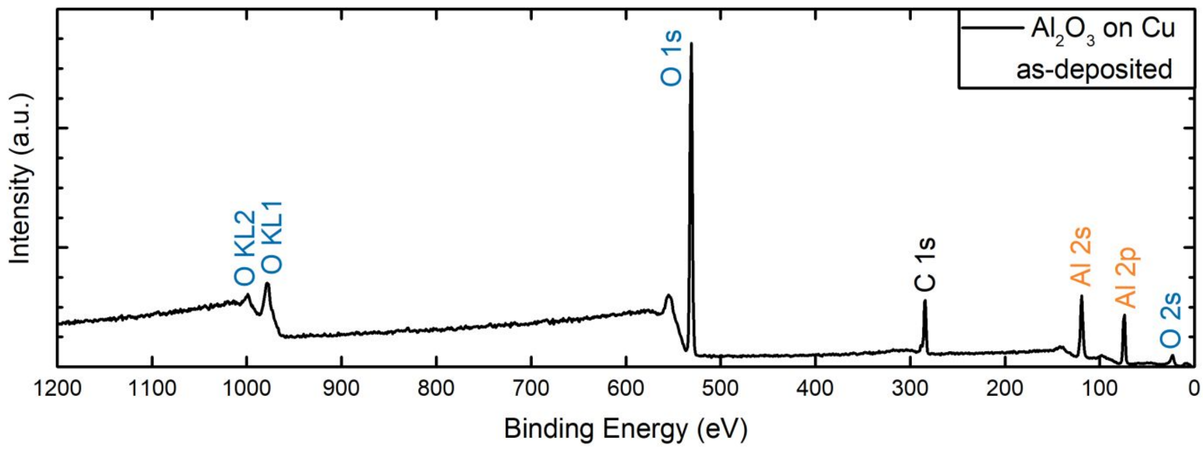

Figure S2. XPS survey spectra for as-deposited Al_2O_3 reference sample. The Al and O atoms were detected on the surface, additional with C which comes from the environment. According to curve fitting of the survey spectra, oxygen was found 47.6 at.% and aluminum was 33.25 at.% which indicates the formation of Al_2O_3 with [Al:O]: 0.6 atomic ratio.

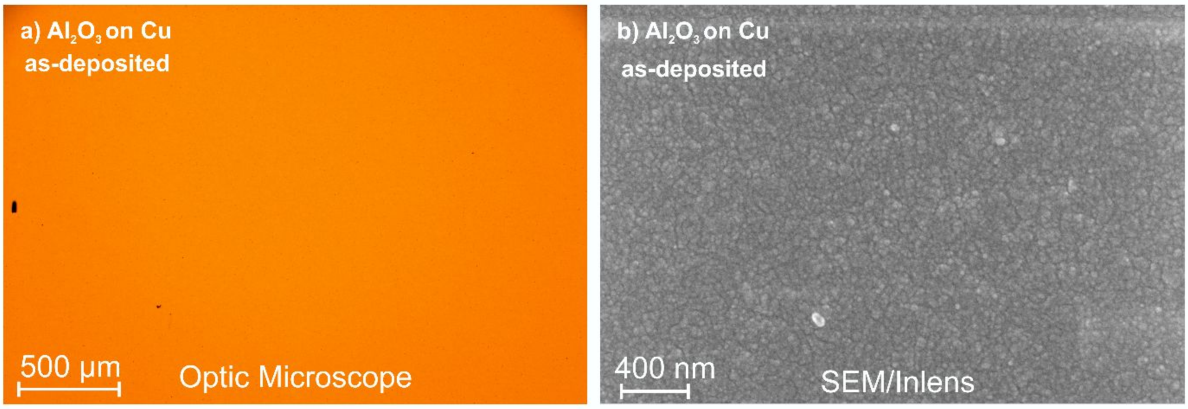

Figure S3. Surface properties of Al_2O_3 (a) optic microscope and (b) SEM before wet etching. The SEM images were taken in 2 keV with an Inlens detector. The reference ALD parameters were used for Al_2O_3 deposition (150 °C, 20 ms, without plasma pretreatment).

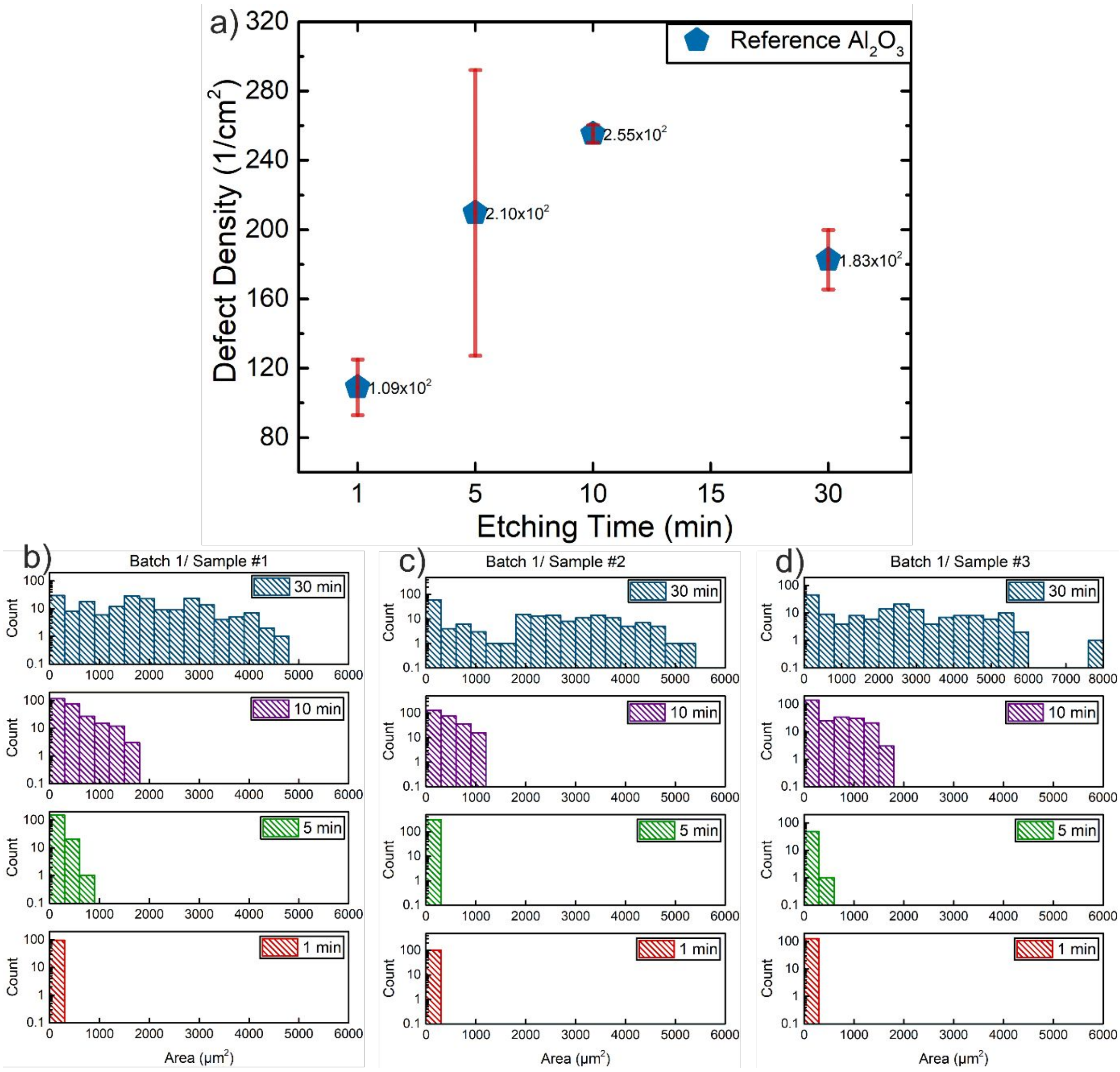

Figure S4. Wet etching time dependency of Al_2O_3 reference sample. (a) Defect density in cm^2 for different etching times. The standard deviations were calculated based on three different samples. The defect size distribution for different etching times is given for (b) sample 1, (c) sample 2, and (d) sample 3. While the defect density was 109 in 1 cm^2, it was getting similar, around 200 1/cm^2, for 5 min, 10 min, and 30 min. According to particle distribution, the smaller defects were getting larger with longer penetration but as well new defects started to reveal on the surface. It shows the longer acid penetration provides the overall defect structure with the existence of all types of defect.

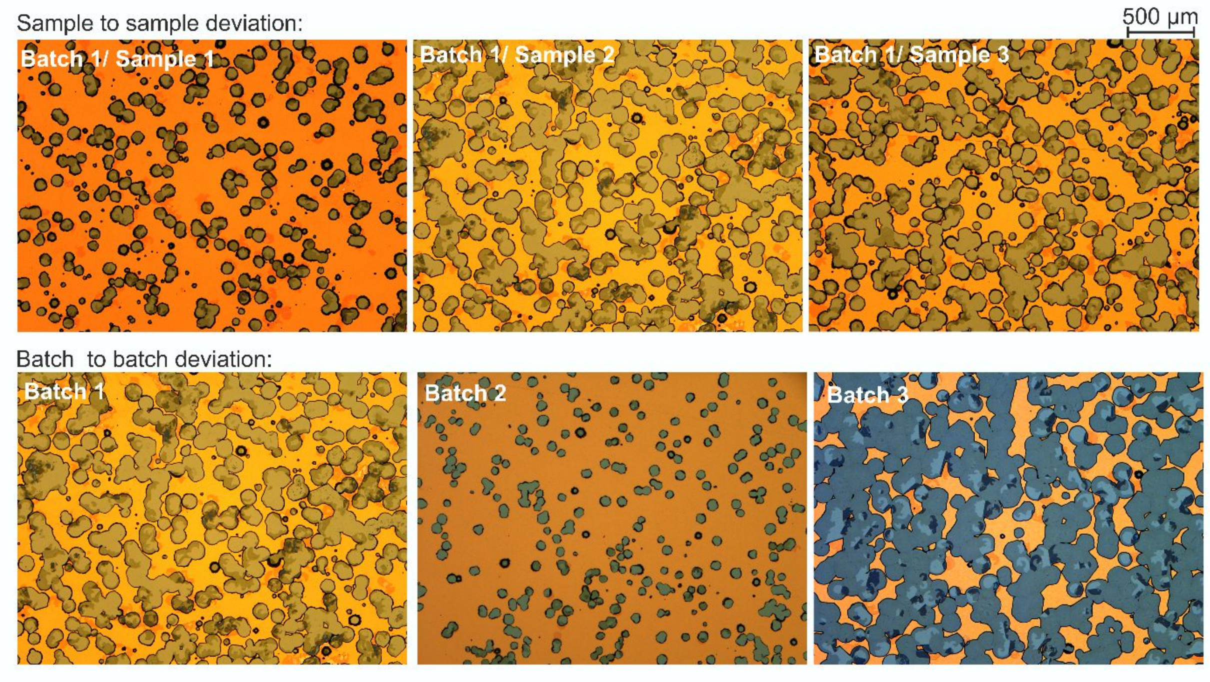

Figure S5. The defect density of reference Al_2O_3 films after 30 min nitric acid etching. While sample to sample deviation was found 39.35±10.51 for batch 1, it was found 17.54±2.02 and 77.92±13.62 for batch 2 and 3, respectively. The overall etched area and standard deviation for reference Al_2O_3 films was found 44.935±27.323.

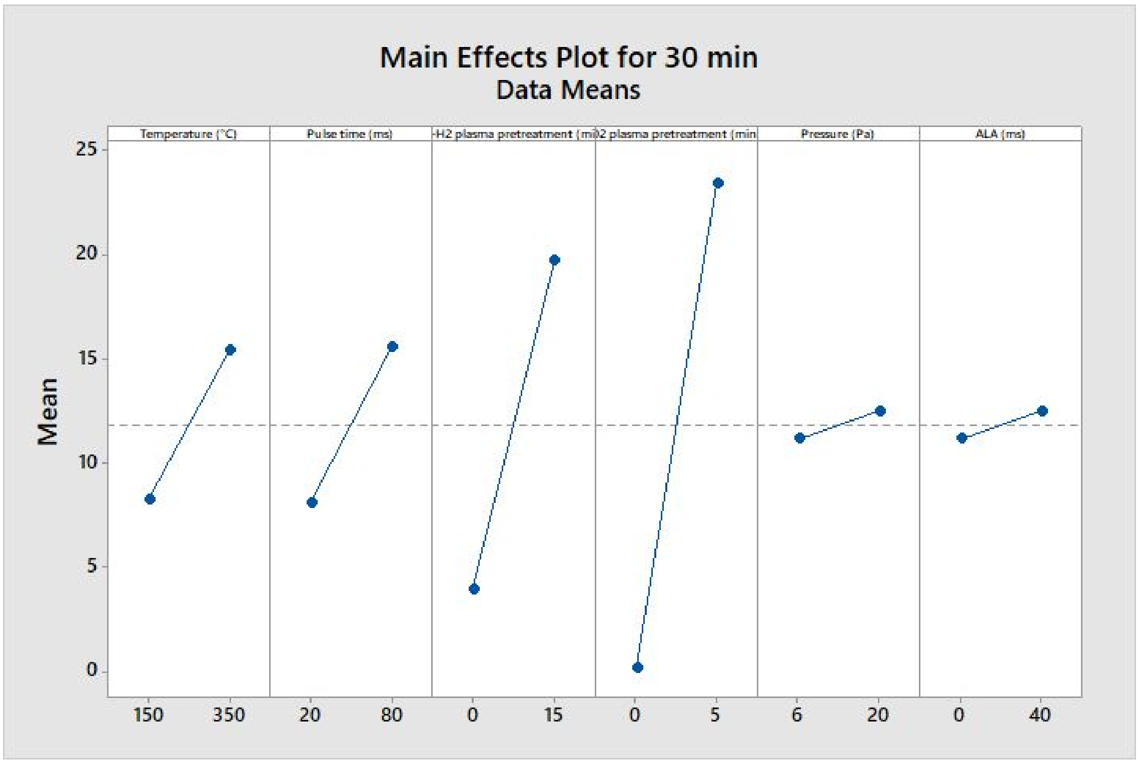

Figure S6. The main effect plot for defect density of Al_2O_3 films according to two-level FFD analysis with six parameters. According to the main effects plot, O_2 plasma considerably affected the defect density but in an antagonistic manner (negative effect of a parameter). The Ar/H_2 plasma pretreatment was also found to have an important effect on defect density. The temperature and pulse time was considered relatively important, and have the same effect magnitude. The pressure and additional waiting time between cycles (ALA) were considered to have a low effect on defect density. While O_2 was found significant, it was omitted from the optimization step due to its undesired effect on defect density.

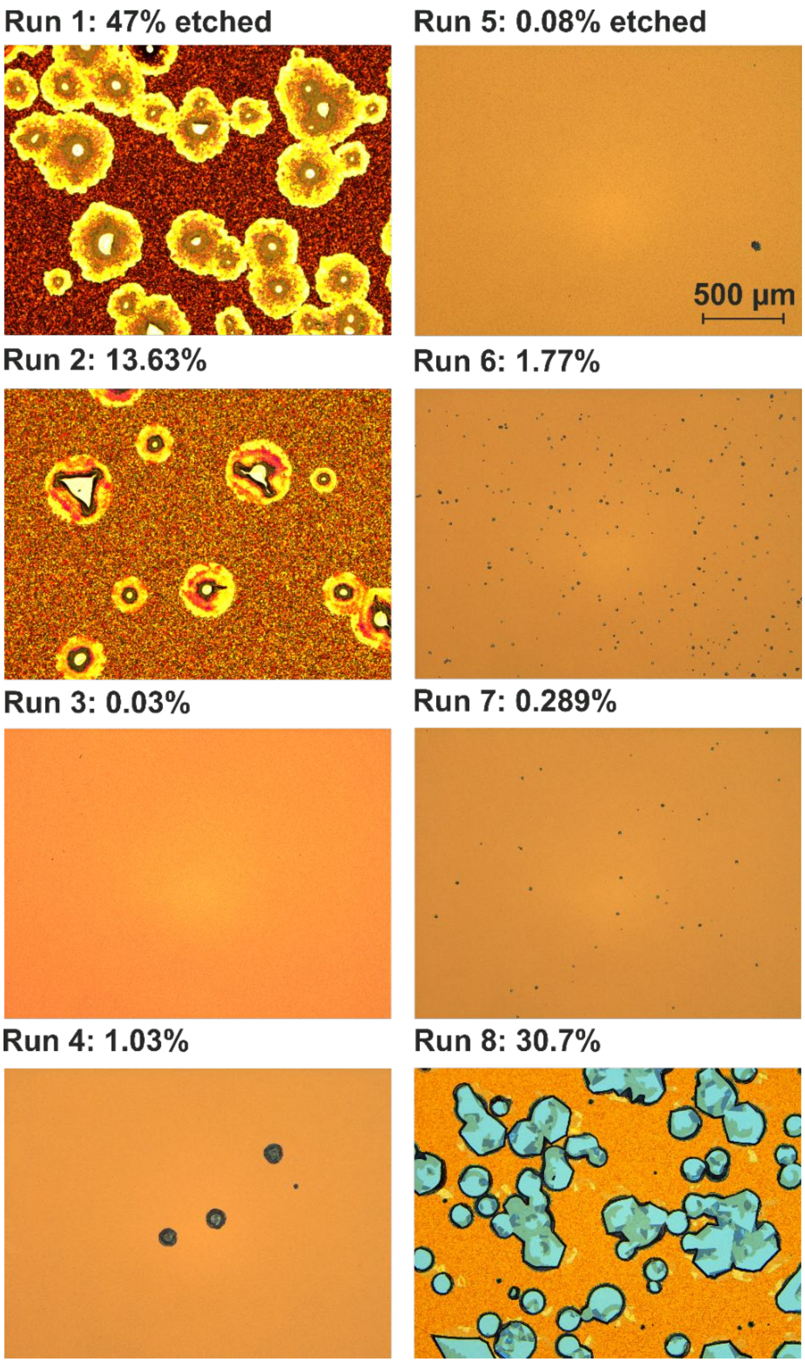

Figure S7. The optic microscope images for 30 min wet etching as a result of two-level screening FFD analysis. All optic microscope images were taken with x5 magnification, as indicated in Run5-500 μm.

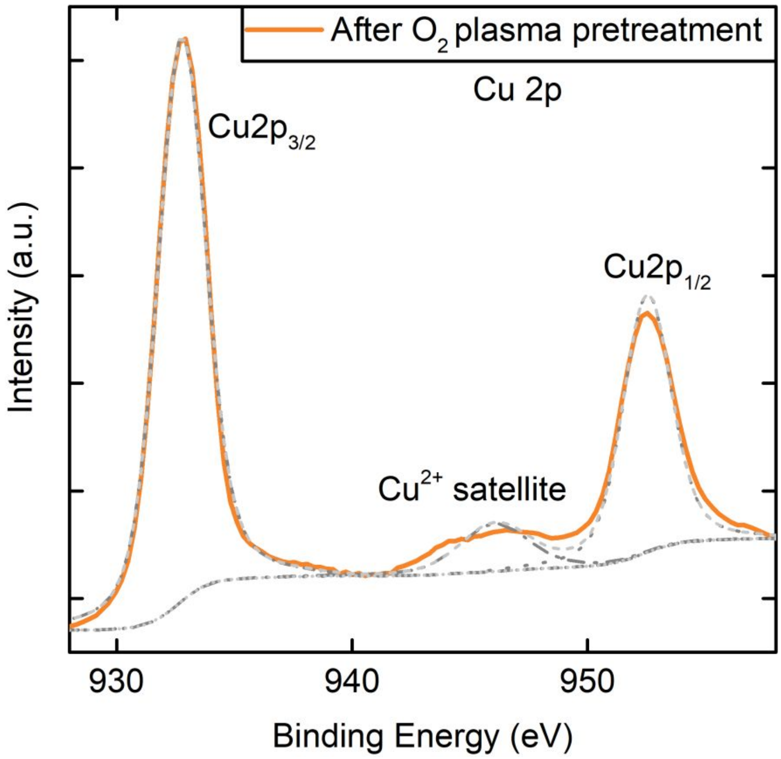

Figure S8. XPS of copper peaks from the surface of O_2 plasma pretreated copper shows the presence of oxide formation. The coper peaks were labeled at 834.5 eV and 954.2 eV and strong Cu^{2+} satellite peak at 944.7 eV which were referenced to copper oxide formation. The O_2 plasma pretreatment was applied at 350 °C for 5 min. According to *in situ* spectroscopic ellipsometry measurements, the copper oxide thickness was measured ~2.5 nm before treatment, and ~155 nm after treatment.

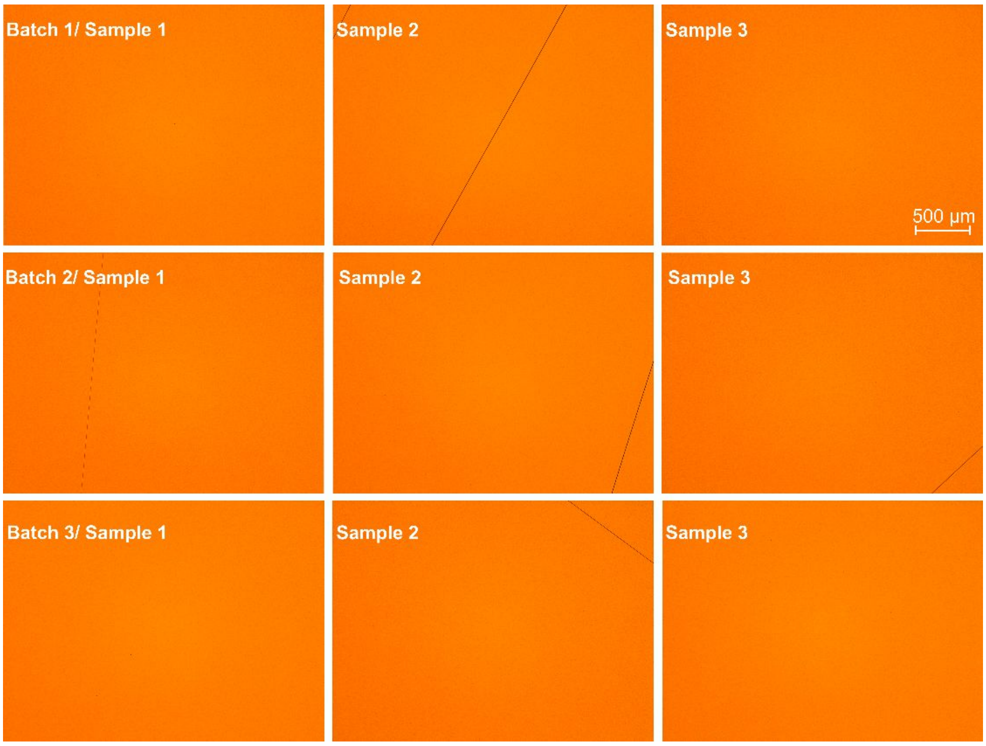

Figure S9. Optic microscope images for repeatability of optimum process parameters (step 3.6) by 30 min wet etching. The same experimental parameters were applied three times (batch deviation) in which 3 samples were placed in each case (sample deviation). The sample to sample deviation was calculated for each batch and indicated as average value and standard deviation. The batch to batch deviation was calculated based on the average value with error propagation analysis. Table S1 summarized the calculated values.

Table S1. Repeatability of optimum state defect density for Al_2O_3 films

	Defect Density (%)	
Batch 1	0.0027	±0.0012
Batch 2	0.0032	±0.0003
Batch 3	0.0037	±0.0017
Batch to Batch	0.0033	±0.0011
Step 3.6	0.0014	±0.0007

Table S2. Additional experiment details that were used during parameters effect analysis in Bayesian Optimization. The experiments were conducted during parameters limit search to identify the low and high levels in FFD analysis. Additionally, Run 3 from FFD analysis was also added as other parameters were in the reference case.

Experiments	Pretreatment	ALD process parameters		Etched Area (%)
	Ar-H_2 plasma (min)	Temperature (°C)	Pulse time (ms)	
1	0	350	80	0.09
2	15	150	20	0.5
3	15	150	80	0.17
4	15	350	80	0.02
Run 3 (FFD)	15	350	20	0.004

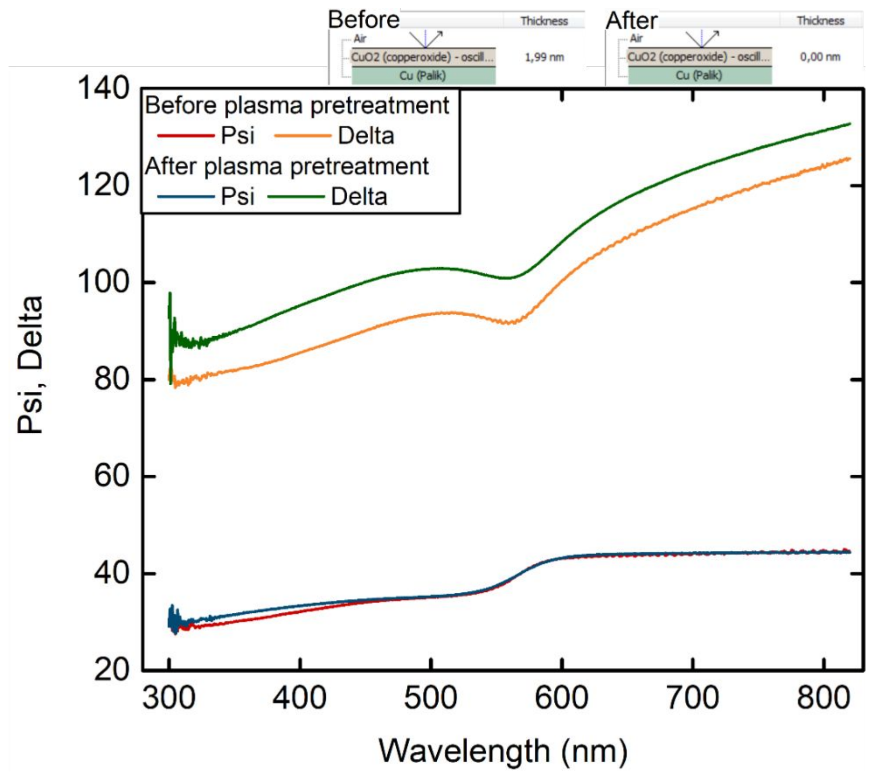

Figure S10. By application of the plasma pretreatment, the air-formed oxide layer was removed according to the *in situ* spectroscopic ellipsometry (SE) data. The psi and delta plot is given as a function of wavelength. Before Ar/H_2 plasma pretreatment, *in situ* SE measurement showed 1.99 nm Cu_2O thickness according to the fitting analysis of psi and delta curves. With the application of 5 min Ar/H_2 plasma pretreatment at 250 °C, the thickness of Cu_2O found zero, indicated by the change of delta curve. For the optical model, copper was defined as the file layer, and Cu_2O layer according to Drude and Lorentz oscillator. The model-fitting results are given on the top of the figure for before and after plasma pretreatment with 1.99 nm and 0 nm Cu_2O thickness, respectively.

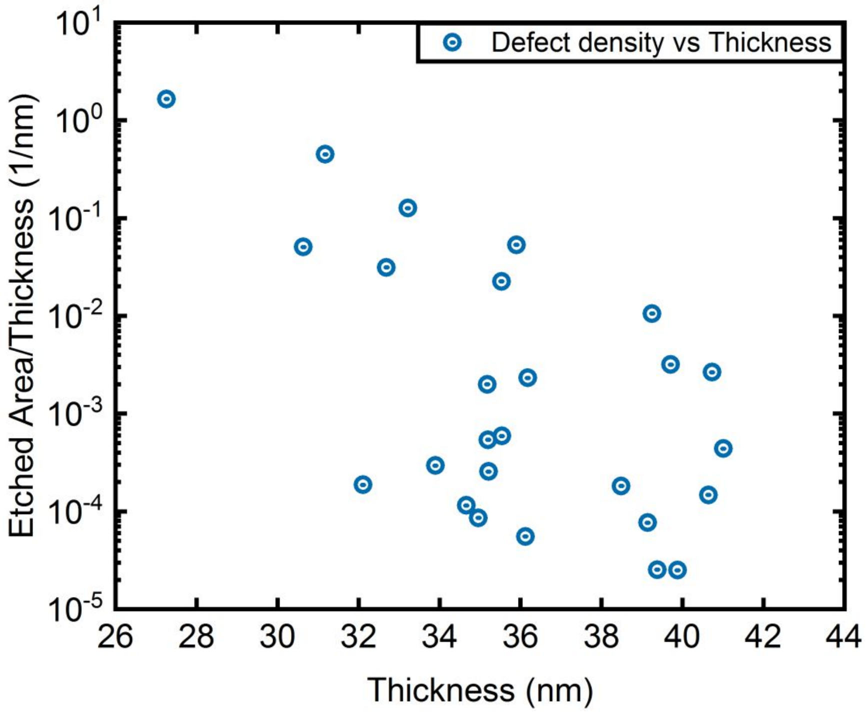

Figure S11. Etched area percentage per thickness (1/nm) for ALD-Al_2O_3 thin films at different process parameters. For the films deposited with optimized parameters, the defect density per deposited thickness (etched area %/nm) does not depend significantly on the thickness. In other words, depositing more material for optimized films does not increase the corrosion protection properties.
